# Supplementary material for: Meta-Analysis of 16S rRNA Sequencing Reveals Altered Fecal but Not Vaginal Microbial Composition and Function in Women with Endometriosis
Source: Medicina (Kaunas). 2025 May 14;61(5):888. doi: 10.3390/medicina61050888 (PMC12112980; doi:10.3390/medicina61050888)
Supplement: Supplementary file 1 [file medicina-61-00888-s001.zip › Figure S1.pdf]

PRISMA 2009 Flow Diagram

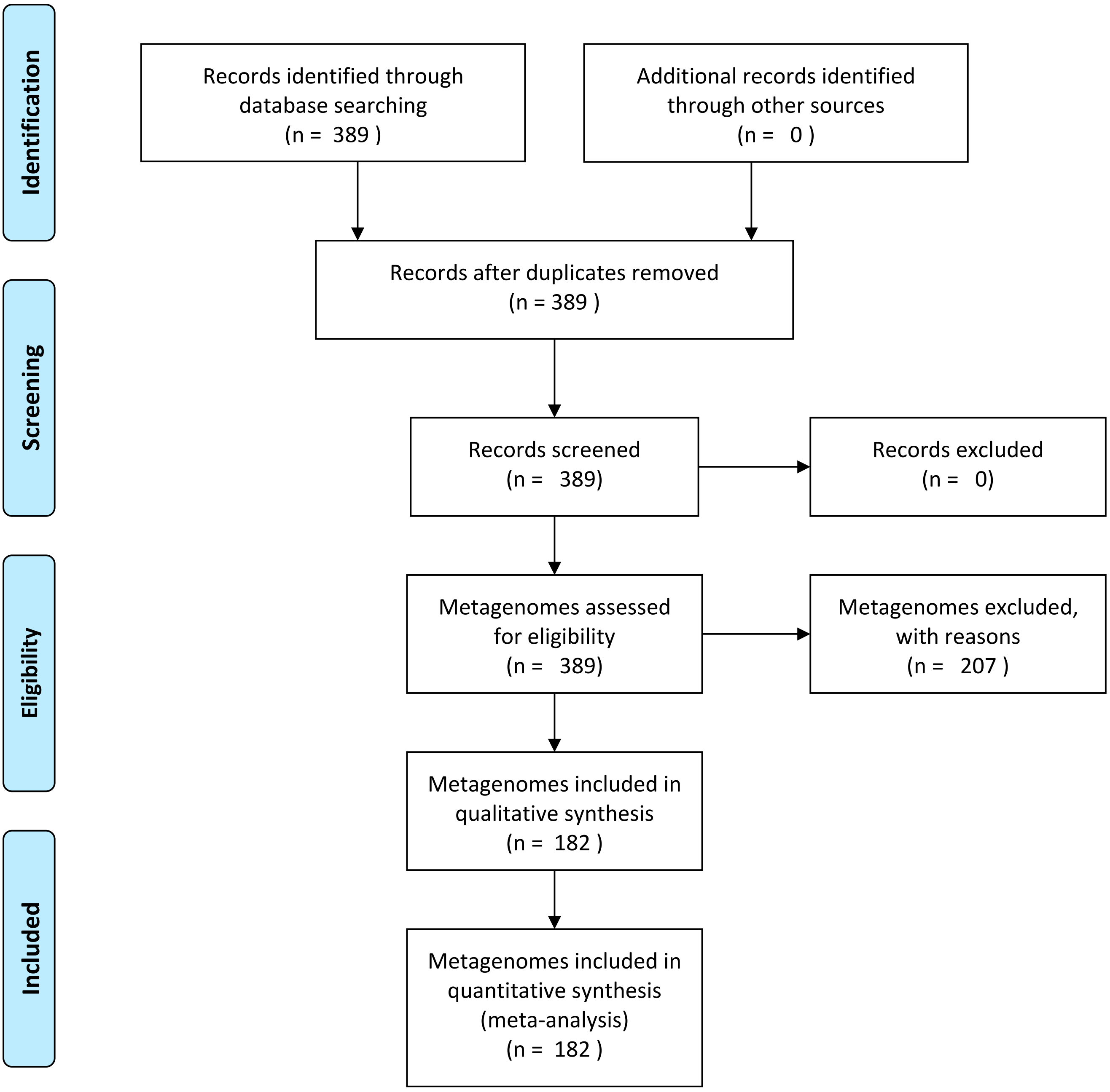

From: Moher D, Liberati A, Tetzlaff J, Altman DG, The PRISMA Group (2009). *P*referred *R*eporting *I*tems for *S*ystematic Reviews and *M*eta-*A*nalyses: The PRISMA Statement. PLoS Med 6(6): e1000097. doi:10.1371/journal.pmed1000097

For more information, visit www.prisma-statement.org.
