# Supplementary material for: Meta-Analysis of 16S rRNA Sequencing Reveals Altered Fecal but Not Vaginal Microbial Composition and Function in Women with Endometriosis
Source: Medicina (Kaunas). 2025 May 14;61(5):888. doi: 10.3390/medicina61050888 (PMC12112980; doi:10.3390/medicina61050888)

Supplementary Figure S2: Relative abundance of top 10 phyla (A) and top 15 genus (B)

A

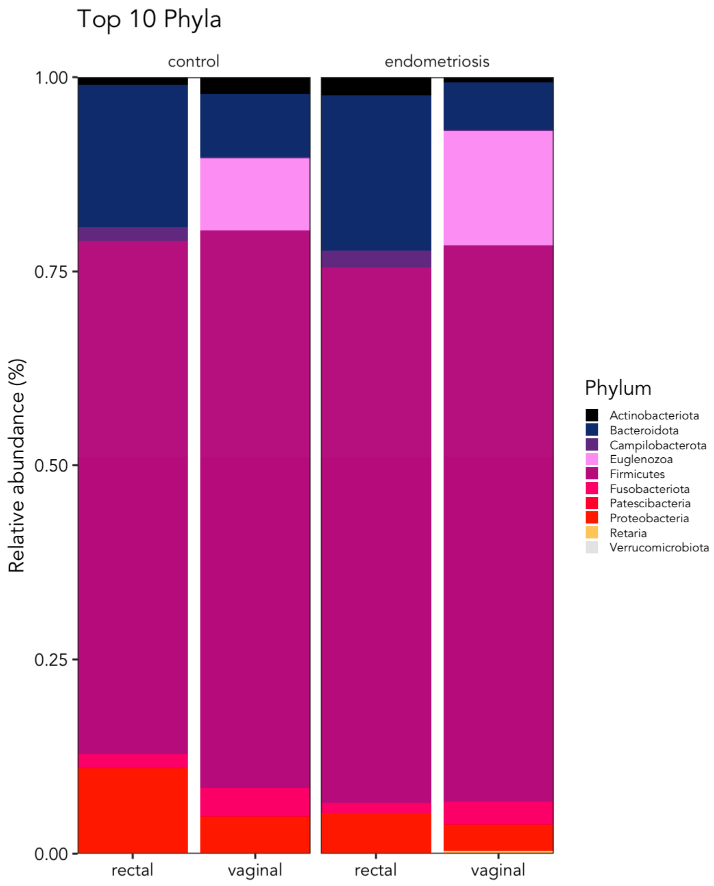

B

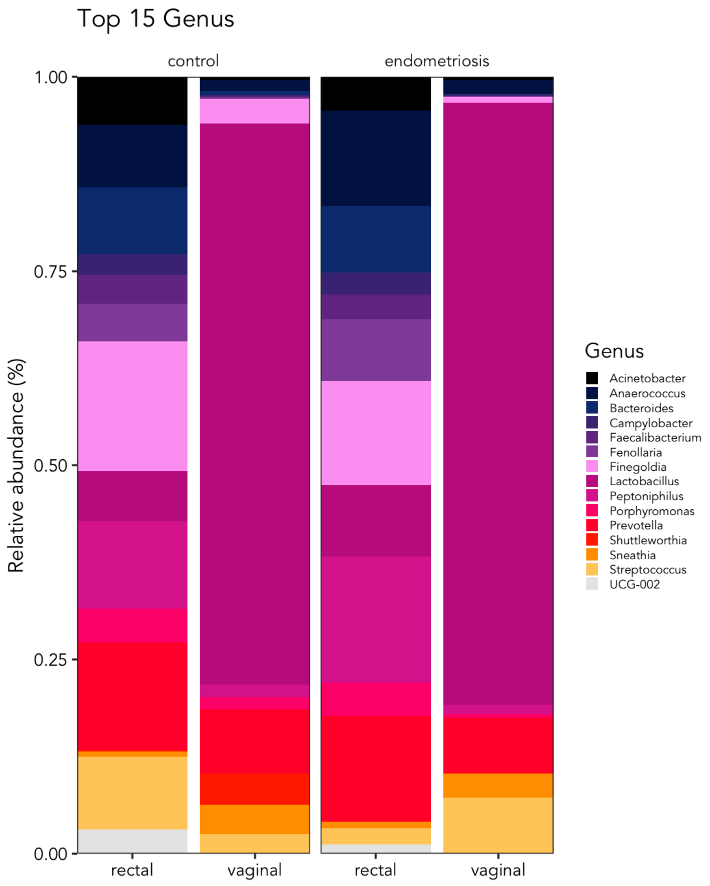

Supplementary Figure 2: Correlation between MEI and host age

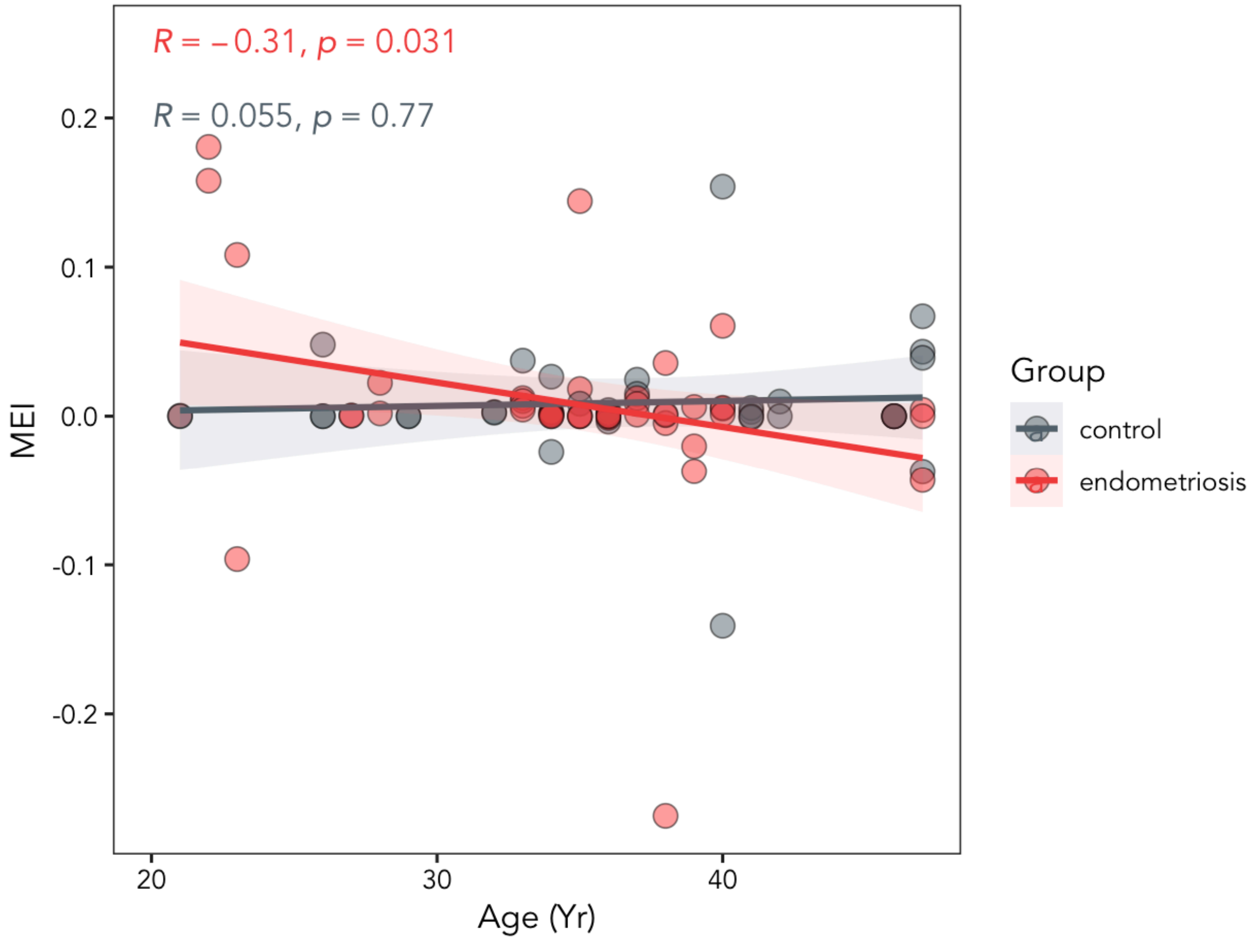

Supplement: Supplementary file 1 [file medicina-61-00888-s001.zip › Figure S2.pdf]
